# In Situ Construction of Near-Infrared Response Hybrid Up-Conversion Photocatalyst for Degrading Organic Dyes and Antibiotics

**DOI:** 10.3390/molecules28186674

**Published:** 2023-09-18

**Authors:** Lianqing Yu, Yankun Wang, Xinhai Su, Chong Liu, Kehui Xue, Huihua Luo, Yaping Zhang, Haifeng Zhu

**Affiliations:** 1 School of Materials Science and Engineering, China University of Petroleum, Qingdao 266580, China; wang_yankun1@upc.edu.cn (Y.W.); buctlc96@163.com (C.L.); kehuixue@163.com (K.X.); huihua_luo@upc.edu.cn (H.L.); 2 School of Chemical and Environmental Engineering, Hanshan Normal University, Chaozhou 521041, China; suxinhai1@hstc.edu.cn; 3 College of Science, China University of Petroleum, Qingdao 266580, China; zhangyp@upc.edu.cn (Y.Z.); zhufeng_97@upc.edu.cn (H.Z.)

**Keywords:** up-conversion, photocatalyst, charge–energy transfer, photodegradation

## Abstract

Unique nonlinear optical properties for converting low-energy incident light into high-energy radiation enable up-conversion materials to be employed in photocatalytic systems. An efficient near-infrared (NIR) response photocatalyst was successfully fabricated through a facile two-step method to load BiOBr on the Nd^3+^, Er^3+^@NaYF_4_ (NE@NYF) up-conversion material. The NE@NYF can transform NIR into visible and UV light and promote charge–energy transfer in the semiconductor. Consequently, the as-obtained photocatalysts exhibit excellent photodegradation performance for rhodamine B dye (RhB) and tetracycline (TC) organic pollutants. About 98.9% of the RhB was decomposed within 60 min with the 20% NE@NYF-B sample, outperforming the pristine BiOBr (61.9%). In addition, the 20% NE@NYF-B composite could decompose approximately 72.7% of the organic carbon during a 10 h reaction, which was almost two-fold more than that of BiOBr. Meanwhile, a possible charge transfer mechanism is proposed based on the recombination of electron–hole pairs and reactive oxygen species. This work provides a rational hybrid structure photocatalyst for improving photocatalytic performance in the broadband spectrum and provides a new strategy for NIR light utilization.

## 1. Introduction

The solar spectrum is composed of 5% ultraviolet (300–400 nm), 43% visible (400–700 nm), and 52% near-infrared (700–2500 nm) radiation [1]. Up-conversion materials can effectively harvest near-infrared (NIR) and harness the broadband spectrum of the solar spectrum. These materials can also be applied for biological imaging, light-triggered drug release, and anti-counterfeit fields [2,3,4,5,6,7] due to their unique nonlinear optical properties for converting low-energy incident light into high-energy radiation. According to the first law of photochemistry, only the light absorbed by the molecules can effectively set off photochemical reactions in the system. However, most photocatalytic materials only absorb ultraviolet light, leading to insufficient sunlight energy utilization [8]. Thus, it is an accessible way to use up-conversion materials to extend the photo-absorption range in visible light, and even infrared light regions, to improve photocatalytic performance [9,10,11,12]. Up-conversion luminescent materials have the property of anti-Stokes luminescence. The can be excited by long-wavelength low-frequency light and can emit short-wavelength high-frequency light [13]. At present, up-conversion luminescence mainly occurs in compounds doped with lanthanide and rare-earth ions. Up-conversion matrix materials include NaYF_4_, NaGdF_4_, and some fluorides, oxides, and sulfides. Among them, NaYF_4_ is an excellent host material for near-infrared responsive phosphors that can emit visible light, with the highest up-conversion luminescence efficiency. After doped lanthanides, it can collect two or more low-energy NIR photons by fluorescence resonance energy transfer (FRET), enabling re-absorption and redevelopment by adjacent UV or visible light-responsive components [11,14,15,16].

According to the second law of photochemistry, an absorbed photon can only activate one molecule. Since the up-conversion material undergoes FRET through a radiative or non-radiative energy transfer mechanism, the surrounding semiconductor can re-absorb this part energy for photo-generated carriers, which is beneficial to increase the generation of photocatalytic reactive species [17]. Therefore, compounding up-conversion material with a suitable semiconductor material is expected to be an effective strategy for improving the energy conversion efficiency of semiconductors.

Notably, BiOX(Br, Cl, I) semiconductor is widely used in photocatalysis and many other fields due to its virtues of low cost, low toxicity, and high efficiency [11,16,17,18,19,20]. For instance, Li et al. [21] developed a ternary composite consisting of Sr_2_LaF_7_:Yb^3+^, Er^3+^ up-conversion nanocrystals and Bi nanoparticle loaded-BiOBr nanosheets with oxygen vacancies (OVs, SLFBB) through a multi-step hydrothermal process and estimated the photodegradation property towards a 10 mg/L BPA solution. Zhang et al. [22] prepared β-NaYF_4_: Pr^3+^, Li^+^ (NYF) with BiOCl photocatalyst, which can be excited by visible light and generate UV light (λ < 280 nm). After combination with BiOCl, the NaYF_4_ generated additional •O_2_^−^ and •OH, and greatly enhanced its photocatalytic activity. Bai et al. [23] designed a NaYF_4_: Yb, Tm-BiOCl nanocrystals hybrid structure based on a suitable BiOCl (110) facet, leading to higher energy transfer efficiency and generating more charge carriers. Li et al. [16] fabricated SrF_2_-Bi_2_O_3_-B_2_O_3_/Yb^3+^, Tb^3+^ with BiOBr via a glass-ceramic etching method. It is proved that the Bi-containing up-conversion glass ceramic is suitable for constructing heterojunctions, which contributes to efficient luminescence. The above composites are limited by their multiple-step and high temperature/pressure procedures. Therefore, it is highly necessary to develop a low-temperature, in situ surface, self-grown procedure to strengthen the interface of the hybrid structure.

In this study, we have developed a new method combining semiconductor and up-conversion materials to construct a 3D-structured Nd^3+^, Er^3+^@NaYF_4_-BiOBr (NE@NYF-B) hybrid photocatalyst. 3D BiOBr has the advantages of high up-conversion luminescence efficiency, dense transport channels for transporting small organic molecules, and easy recycling ability. To our knowledge, such a method has not been reported previously. Additionally, the micro-hexagonal up-conversion material NE@NYF was prepared by the hydrothermal method, in which neodymium (Nd^3+^) was used as a sensitizer and erbium (Er^3+^) as an activator. Then BiOBr nanosheets were loaded on the surface of NE@NYF through the in situ surface, self-grown strategy. The NE@NYF up-conversion material could transform NIR into visible and UV light, which promote the charge–energy transfer inside the semiconductor, and the constructed structure reduces the migration distance of photo-generated carriers to accelerate the electron transfer. The photocatalytic efficiency and apparent reaction rate constant of the NE@NYF-B composite were improved by 212% and 168%, respectively, compared with the original BiOBr. Moreover, the effective acting radicals are further analyzed in detail based on trapping experiments to explore the possible mechanism. In practical experiments, a certain proportion of composite catalyst is added for degradation according to the concentration of pollutants. And the catalytic reaction process will produces some active substances with oxidizing properties to degrade the pollutants into non-toxic small molecules without secondary pollution. The purified composite material can be removed from water by filtration or magnetic flocculation. This work demonstrates that the NIR light utilization strategy of up-conversion materials can be used for high-efficiency photodegradation.

## 2. Result

### 2.1. Structure and Morphology Characterizations

NE@NYF was first prepared in a Teflon-lined stainless-steel autoclave at 200 °C for 18 h.

**Figure 1:**
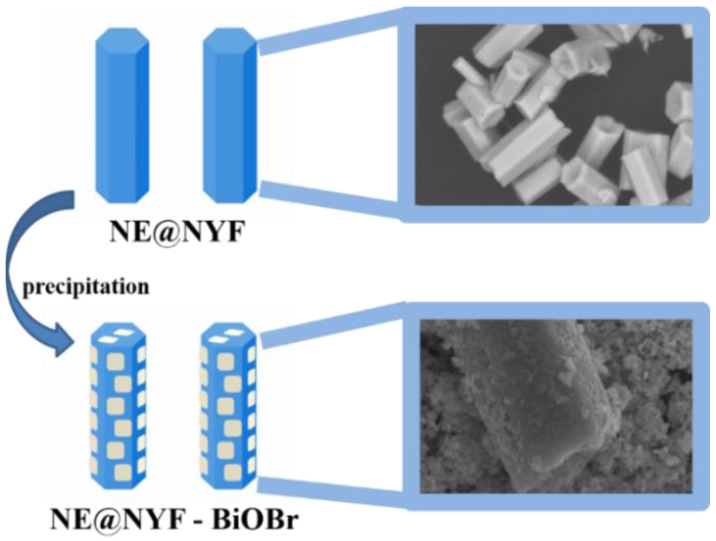
Schematic diagram of synthesis of Nd^3+^ and Er^3+^@NYF-BiOBr.

**Figure 2:**
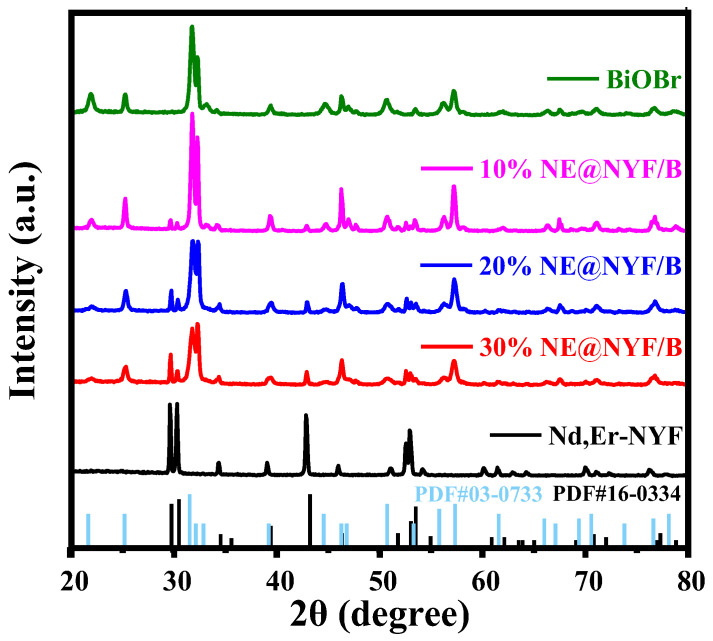
XRD patterns of NE@NYF-B samples with different ratios.

Then NE@NYF-BiOBr was prepared by a co-precipitation method in 25 mL EG solutions, with molar ratio of NE@NYF to BiOBr of 10–30%, as shown in Figure 1. XRD was used to study the crystallographic structure characteristics of samples. The XRD patterns of NE@NYF, NE@NYF-BiOBr, and pristine BiOBr are displayed in Figure 2. It is worth noting that the positions and intensities of peaks of NE@NYF correspond well to the phase of NaYF_4_ (PDF#16-0334), and no additional peaks are observed. The BiOBr peak also corresponds well to BiOBr (PDF#03-0733). The narrow peaks with high intensity also indicate the good crystallinity. In the synthetic procedure, Nd(NO_3_)_3_∙5H_2_O and Er(NO_3_)_3_∙5H_2_O are precursors to modify NYF. Therefore, Nd and Er can replace the Y sites in the NYF lattice, forming the NE@NYF structure during the hydrothermal reaction. After doping, the XRD peaks of NE@NYF reveal an inconspicuous shift to lower 2*θ* angels, indicating the occurrence of lattice shrinkage. According to Bragg’s Law:

2dsinθ=nλ

where *d* is the interplanar crystal spacing, *θ* is the angle between the incident X-ray and the corresponding crystal plane, *λ* is the wavelength of the X-ray, and *n* is the diffraction order. The radius of Nd^3+^ (99.5 pm) is bigger than that of Y^3+^ (89.3 pm), while the radius of Er^3+^ (88.1 pm) is similar to that of Y^3+^, so Er^3+^ causes no significant influence on the peaks of NYF. According to the above analysis, the NE@NYF and NE@NYF-BiOBr are successfully synthesized through hydrothermal method, and the crystal structure of the material does not change after loading with BiOBr. Moreover, the intensity of the NE@NYF peaks decreases as the ratio of NE@NYF increases.

**Figure 3:**
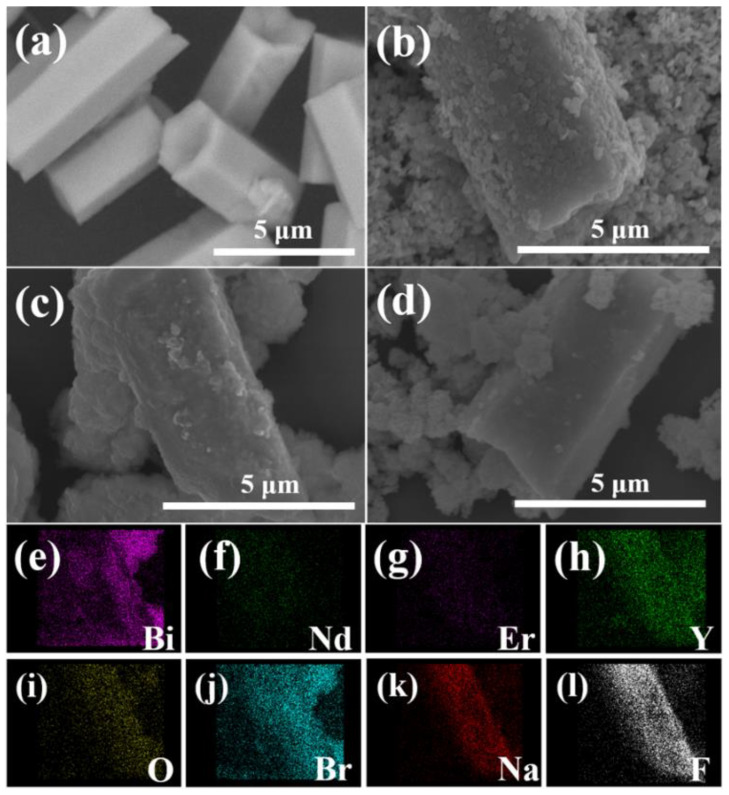
SEM images of (**a**) 5% NE@NYF-B, (**b**) 10% NE@NYF-B, (**c**) 20% NE@NYF-B, (**d**) 30% NE@NYF-B and the corresponding elements distribution mapping of 20% NE@NYF-B: (**e**) Bi, (**f**) Nd, (**g**) Er, (**h**) Y, (**i**) O, (**j**) Br, (**k**) Na, and (**l**) F.

**Figure 4:**
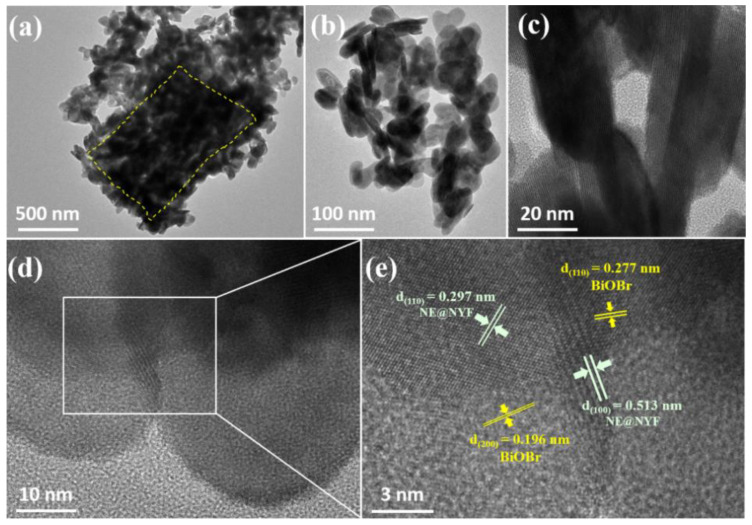
(**a**,**b**) TEM images and (**c**–**e**) HRTEM images of 20% NYF-B composite, yellow dotted box is BiOBr.

The morphologies and element distributions of NE@NYF and NE@NYF-BiOBr were analyzed by SEM and TEM, and the results are shown in Figure 3 and Figure 4. NE@NYF presents hexagonal micro-rods with lengths of about 5–7 μm and diameters of about 2–3 μm. The surface of NE@NYF is rather smooth, while many nanoparticles can be clearly seen after loading BiOBr on the surface of NE@NYF, implying the successful fabrication of NE@NYF-B. As the proportion of NE@NYF increases, the BiOBr nanoparticles on the surface are relatively reduced. Element mappings were used to determine the distribution of elements in the composite of 20% NE@NYF, as shown in Figure 3e–l, which exhibits homogeneous distributions of Nd, Y, Bi, Br, and so forth.

The HRTEM of 20% NYF-BiOBr (Figure 4) reveals a clear and continuous lattice fringe of the composite. The interplanar crystal spacing measured at approximately 0.513 nm and 0.297 nm can be ascribed to (100) and (110) facets of up-conversion NE@NYF material, while the d-spacing of 0.196 nm and 0.282 nm are (200) and (102) facets of BiOBr. The SEM and TEM images combined with XRD analysis demonstrate the formation of the NE@NYF-B composites between the up-conversion material and BiOBr.

### 2.2. XPS Analysis

**Figure 5:**
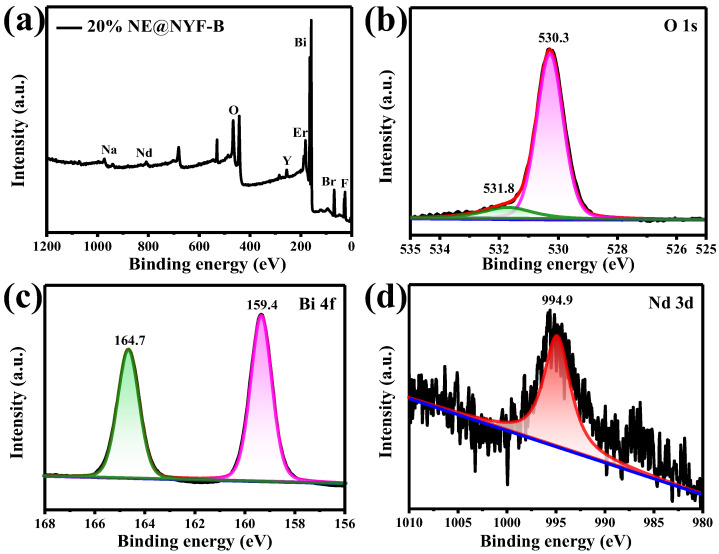
XPS spectra of the 20% NE@NYF-B (**a**) survey, (**b**) Bi 4f, (**c**) O 1s, and (**d**) Nd 3d.

XPS was conducted to further investigate the chemical compositions and elemental valence states in the composite of 20% NE@NYF-B, as shown in Figure 5. The survey scan XPS spectrum confirms the existence of the Nd/Er/Y/Bi/O/Br elements in the composite (Figure 5a). Figure 5b–d illustrate the O 1s, Bi 4f, and Nd 3d core-level XPS spectra, respectively, with high resolution. The O 1s peak can be separated into two main peaks positioned at 530.3 and 531.8 eV, which are assigned to the crystal lattice oxygen from Bi-O bonds and the chemisorbed oxygen from the hydroxyl-like group [17], respectively. The appearance of two sharp peaks at 159.4 and 164.7 eV correspond to the binding energies of Bi 4f_7/2_ and Bi 4f_5/2_ for the Bi 4f XPS spectrum, suggesting the existence of a Bi^3+^ oxidation state [24,25,26]. The peak at 994.9 eV in Figure 5d comes from Nd^3+^. These results further confirm that the composite has been prepared successfully.

### 2.3. Optical Properties

**Figure 6:**
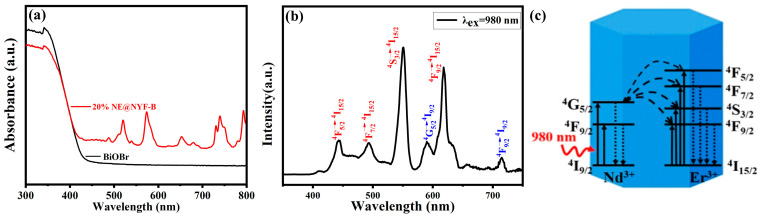
(**a**) UV–vis spectra of BiOBr and 20%NE@NYF-B. (**b**) Up-conversion luminescence spectra of 20% NE@NYF-B (λ_ex_ = 980 nm). (**c**) Possible energy transfer mechanism.

The light absorption characteristics of the sample before and after the composite up-conversion are investigated in the UV–vis absorption spectrum (Figure 6a). It was found that the light absorption threshold of single component BiOBr is about 430 nm, while after the up-conversion material is compounded, the sample begins to show an absorption peak in the range of more than 500 nm, indicating that the light absorption of the 20% NE@NYF-B composite sample expands to the infrared region. This phenomenon contributes to an improvement in the efficiency of the use of sunlight and accelerates the energy conversion.

The room-temperature up-conversion luminescence spectrum of 20% NE@NYF-B excited under a 980 nm laser is shown in Figure 6b. Nd^3+^ and Er^3+^ are two different luminous centers. The energy absorbed by Nd^3+^ is transferred to Er^3+^ through interaction, with the result that the luminescence of the latter center strengthens. The sample exhibits six distinct NIR luminescence peaks in the range of 350–800 nm. The peaks at 590 nm and 715 nm are attributed to the ^4^G_5/2_→^4^I_9/2_ and ^4^F_9/2_→^4^I_9/2_ transitions of Nd^3+^, respectively [27,28,29,30]. The peaks at 488 nm, 493 nm, 550 nm, and 618 nm can be assigned to ^4^F_5/2_→^4^I_15/2_, ^4^F_7/2_→^4^I_15/2_, ^4^S_3/2_→^4^I_15/2_, and ^4^F_9/2_→^4^I_15/2_, respectively. These peaks suggest there are efficient energy transfers between Nd^3+^ and Er^3+^ (Figure 6c) [29,31,32]. Importantly, with the greatly enhanced visible light emissions in the up-conversion luminescence spectra, 20% NE@NYF-B can absorb more photons than is expected and exhibit much higher photocatalytic activity than pure BiOBr.

### 2.4. Photocatalytic Performance

**Figure 7:**
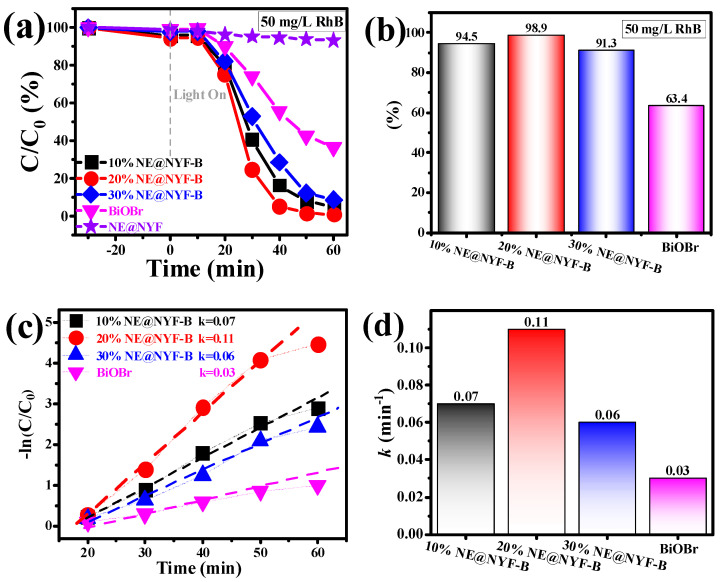
(**a**,**b**) Photocatalytic degradation of 50 mg/L RhB, and (**c**,**d**) the apparent reaction rate constant of samples.

The photocatalytic degradation performance of the synthesized photocatalysts was evaluated under stimulating sunlight irradiation of a 300 W Xe lamp using an initial concentration of RhB at 50 mg/L. The samples and contaminants were first stirred in the dark for 30 min to reach adsorption–desorption equilibrium. Before irradiation, the adsorption of RhB was highest for 20% NE@NYF-B, and 98.9% of the RhB was decomposed within 60 min (Figure 7a). The photocatalytic activity of the NE@NYF-B samples (>90%) was superior to that of the pristine BiOBr (63.4%, Figure 7b). To estimate the photocatalytic capability of the samples, the apparent reaction rate constant (*k*) of the photocatalyst is used to fit the experimental data, as shown in Figure 7c,d. The linear plot of −*ln*(*C*/*C*_0_) vs. illuminated time (*t*), suggests that the photodegradation of RhB followed a pseudo-first-order process. Obviously, the kinetics rate constants of BiOBr, 10% NE@NYF-B, 20% NE@NYF-B and 30% NE@NYF-B are 0.03 min^–1^, 0.07 min^−1^, 0.11 min^−1^ and 0.06 min^−1^, respectively. The value of *k* improved from 0.03 min^−1^ for pristine BiOBr to 0.11 min^−1^ for 20% NE@NYF-B, which is attributed to the superior light absorption ability and utilization capacity to enhance photocatalytic oxidation performance. Nevertheless, an excess ratio of NE@NYF could cover up the active surface of BiOBr, resulting in a decrease in catalytic activity.

**Figure 8:**
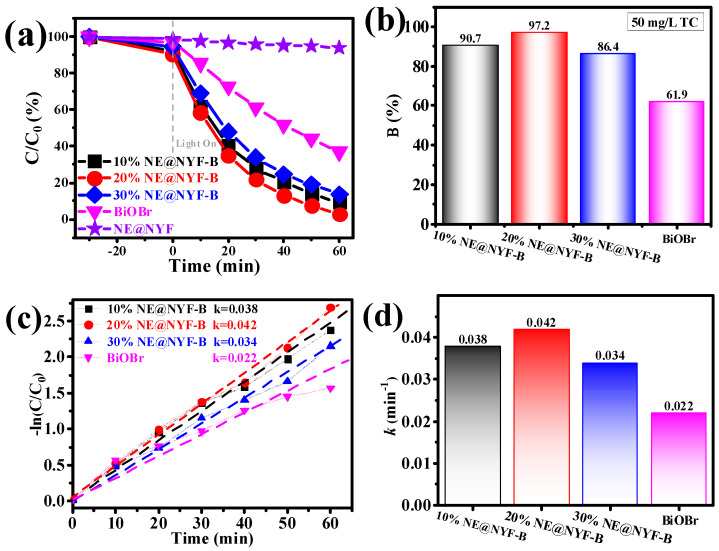
(**a**,**b**) Photocatalytic degradation of 50 mg/L TC, and (**c**,**d**) the apparent reaction rate constant of samples.

The photocatalytic degradation performance of TC with an initial concentration of 50 mg/L is exhibited in Figure 8. As expected, all the NE@NYF-B samples performed better than the original BiOBr (61.9%). And 20% NE@NYF-B achieved a degradation of 97.2% TC within 60 min, which is 1.57 times higher than that of pristine BiOBr (Figure 8a,b). From Figure 8c, the apparent reaction rate constant *k* is calculated. It is important to note that the *k* of 20% NE@NYF-B sample is about two times higher than that of the pristine BiOBr (Figure 8d). In a word, the synergistic effect of the up-conversion phenomenon to excite the BiOBr semiconductor enhances the absorption ability of light, accelerates the separation of photogenerated carriers, and greatly promotes the photodegradation ability of the composite system. Meanwhile, the combination of up-conversion and semiconductor properties offers new prospects for further development of photocatalysts using broadband spectroscopy.

**Figure 9:**
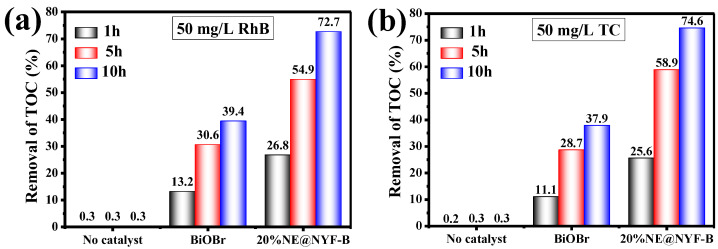
TOC content after photodegradation of (**a**) 50 mg/L RhB; and (**b**) 50 mg/L TC.

For a better evaluation of the photodegradation performance of composites, a TOC (total organic carbon) test was utilized, as shown in Figure 9. The TOC after degradation is sharply decreased with the addition of catalysts. As shown in Figure 9a, pure RhB has no obvious decomposition under light, so its self-decomposition factors are excluded. BiOBr degrades less than 50% of the organic carbon after a 10 h reaction, whereas the composite 20% NE@NYF-B shows excellent photodegradation ability, decomposing approximately 72.7% organic carbon. Similar results were observed for the photocatalytic reaction of TC (Figure 9b). After 10 h of degradation, only 37.9% organic carbon is removed by pure BiOBr in the system, whereas 74.6% is removed by 20%NE@NYF, which is almost two-fold that of BiOBr.

### 2.5. Conductivity and Charge Transfer Efficiency

**Figure 10:**
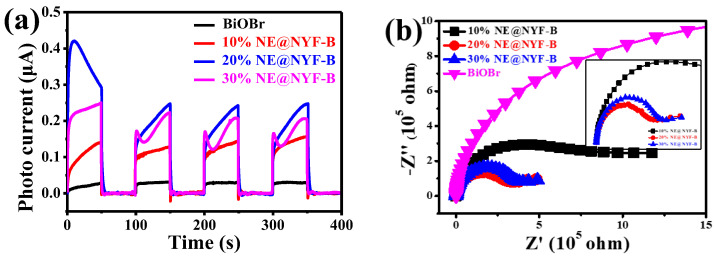
(**a**) Photocurrent response of the samples under irradiation (AM 1.5G), and (**b**) EIS.

Photoelectrochemical measurement is one of the important ways to estimate the photoelectrochemical photocatalytic capability and recombination rate of photo-generated carriers during a photochemical process [33]. The transient photocurrent diagram and electrochemical impedance spectroscopy (EIS) for NE@NYF and NE@NYF-B under irradiation are shown in Figure 10. Under irradiation (AM 1.5 G), the photocurrent values of the composite samples are all higher than that of BiOBr, demonstrating that NE@NYF-B produces more stable electrons, which is beneficial to the production and transition of holes and electrons. Furthermore, the 20% NE@NYF-B shows the highest photocurrent, which is much higher than that of pristine BiOBr (Figure 10a). EIS was utilized to investigate interfacial charge migration dynamics in photocatalytic activity (Figure 10b). A smaller arc radius indicates a lower interfacial resistance, implying the acceleration effect of NE@NYF on charge transmission [34,35]. The existence of NE@NYF enhances the electron transfer of BiOBr and light absorption to improve the photodegradation performance.

## 3. Discussion

**Figure 11:**
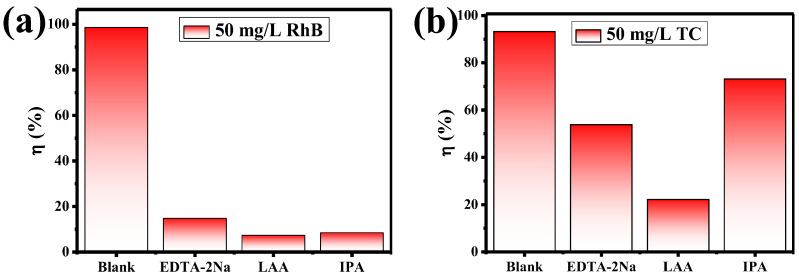
Quenching experiment of 20% NE@NYF-B (Blank for no trapping agent, EDTA-2Na for h^+^, LAA for •O_2_^−^, IPA for •OH): (**a**) in RhB; and (**b**) in TC.

The mechanism of the photocatalytic reaction process was further explored through free radical trapping experiments. Ethylene diamine tetra-acetic acid disodium salt (EDTA-2Na), L-ascorbic acid (LAA), and isopropanol (IPA) were used as scavengers to detect the hole (h^+^), superoxide radicals (•O_2_^−^) and hydroxyl radical (•OH), respectively. As can be seen in Figure 11a, the photodegradation activity of 20% NE@NYF-B reduces significantly with the addition of the three trapping agents, suggesting that h^+^, •O_2_^−^ and •OH synergistically participated in the photodegradation of RhB (Equation (4)). In the trapping experiments of TC (Figure 11b), the photocatalytic activity of 20% NE@NYF-B shows little decrease with the addition of EDTA-2Na and IPA, whereas it reduces starkly with the presence of LAA, indicating that •O_2_^−^ is the primary oxidative species (Equation (4)).

**Figure 12:**
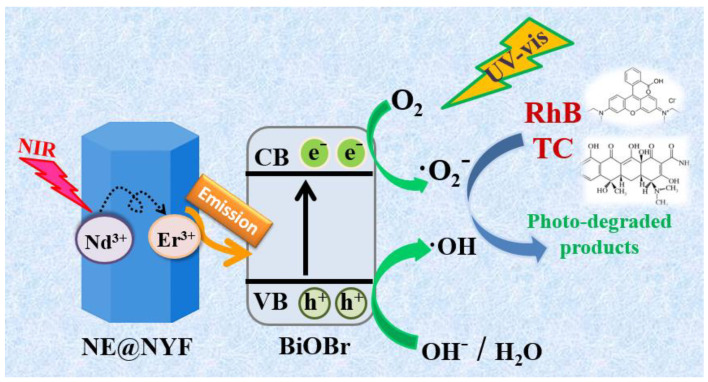
Photocatalytic mechanism of the NE@NYF-B.

Based on the experimental results described above and the previous literature [4,32], a schematic photocatalytic mechanism under irradiation of 20% NE@NYF-B is proposed in Figure 12. Different energy transfer processes could occur on the 20% NE@NYF-B interfaces. With the formation of the excited state in the up-conversion materials, the energy transfer between the interfaces activates the outer BiOBr nanoparticles. Subsequently, the photo-induced carriers migrate and bring about the free radicals. In NE@NYF, Nd^3+^ as a sensitizer can shift the absorbed energy to the activator Er^3+^, which leads to diverse transitions between different energy levels of Er^3+^. Hence, the NIR light with high penetrability from solar light excites the up-conversion material NE@NYF and emits UV and visible light [36,37,38]. In addition, NE@NYF and BiOBr are in close contact, and the energy transfer from NE@NYF to BiOBr contributes to the separation of photogenerated carriers in BiOBr. Additionally, the up-converted light also activates BiOBr via the FRET process, thereby generating electrons and holes (Equation (1)). Meanwhile, O_2_ is trapped by photogenerated e^−^ and reduced to •O_2_^−^ radicals (Equation (2)), and the h^+^ interacts with OH groups on the surface to produce •OH radicals (Equation (3)). BiOBr also absorbs UV photons from solar light and generates photo-carriers due to its intrinsic characteristics. These electrons and holes then migrate to the surfaces and take part in the photocatalytic degradation process with free radicals which degrades RhB and TC. As a result, the h^+^, •O_2_^−^ or •OH can directly oxidize the RhB and TC to small molecules and even to H_2_O and CO_2_ (Equation (4)). The photocatalytic performance of 20% NE@NYF-B was compared with the reported photocatalytic (Table 1). The table shows the corresponding degradation capacities of the different catalysts, and the as-prepared samples exhibit more effective photocatalytic performance and mineralization ability. This work holds significant promise for achieving wastewater purification and access to energy.

(1) $$BiOBr+hv\to e^{-}+h^{+}$$

(2) $$e^{-}+O_{2}\to {•O}_{2}^{-}$$

(3) $$h^{+}+{OH}^{-}\to •OH$$

(4) $$h^{+},\;{•O}_{2}^{-},\;•OH+RhB/TC\to {CO}_{2}+H_{2}O/small\;molecule$$

## 4. Materials and Methods

### 4.1. Materials

All the reagents were used as received without further purification, and deionized water was used throughout all the experiments. Bi(NO_3_)_3_∙5H_2_O (A.R., Aladdin, Bay City, MI, USA), KBr (99.5%, Aladdin), Y(NO_3_)_3_∙5H_2_O (A.R., Aladdin), Nd(NO_3_)_3_∙5H_2_O (A.R., Aladdin), Er(NO_3_)_3_∙5H_2_O (A.R., Aladdin), NaF (A.R., Tianjin Hongyan Chemical Reagent Factory, Tianjin, China), EDTA-2Na (A.R., Tianjin ZhiYuan Reagent Co., Ltd., Tianjin, China), Ethylene glycol (EG, A.R., Shanghai Titan Scientific Co., Shanghai, China).

### 4.2. Materials and Methods

#### 4.2.1. Synthesis of Nd^3+^, Er^3+^@NaYF_4_

A typical method was employed for the preparation of Nd^3+^, Er^3+^@NaYF_4_ (NE@NYF) [35]. In the preparation process, Y(NO_3_)_3_∙5H_2_O, Nd(NO_3_)_3_∙5H_2_O, and Er(NO_3_)_3_∙5H_2_O were dissolved in 30 mL of deionized water at concentrations of 0.5 M, 0.5 M, and 0.1 M, respectively. Subsequently, 1.169 g EDTA-2Na was added to the above solution and stirred violently for 1 h. After that, 20 mL of deionized water containing 1.344 g NaF was added with 1 h of mixing. Finally, the precursor was transferred into a Teflon-lined stainless-steel autoclave then heated in an oven at 200 °C for 18 h. Afterward, the precipitate (labeled NE@NYF) was collected by filtration, washed by deionized water and ethanol, and then dried at 60 °C for 12 h.

#### 4.2.2. Synthesis of Nd^3+^, Er^3+^@NaYF_4_-BiOBr

NE@NYF-BiOBr was prepared by a co-precipitation method at room temperature. First, 0.282 g (1 mmol) of the as-prepared NE@NYF and 0.063 g KBr were dissolved into 25 mL of deionized water. Meanwhile, 0.257 g Bi(NO_3_)_3_∙5H_2_O was dissolved into 25 mL EG by ultrasonic treatment for 10 min. The above solutions were mixed and stirred for 12 h to fully react. Finally, the samples were centrifugally collected after washing through deionized water and ethanol (labeled 20% NE@NYF-B). The molar ratios of NE@NYF to BiOBr at 10%, 20% and 30%, were also prepared to study the effects on microstructure and catalytic performance.

Pure BiOBr was prepared in the same way as mentioned above except for the addition of NE@NYF.

### 4.3. Characterization

The crystalline phases were characterized by X-ray powder diffraction (XRD) at room temperature with Cu Kα radiation in the 2*θ* range of 20–80°. Scanning electron microscopy (SEM, TM-4000) and transmission electron microscopy (TEM, JEOL, JEM-2100) were used to obtain the morphology, and lattice fringe information of the samples. The elements distribution of the sample was obtained by the energy dispersive instrument (EDS). X-ray photoelectron spectra (XPS) were operated on an X-ray photoelectron spectrometer (Thermo Scientific, Waltham, MA, USA). The UCL spectrum was recorded by Edinburgh FLS980. The total organic carbon test (TOC) was characterized by a TOC-L device (Vahimadzu).

### 4.4. Photoelectrochemical Measurements

The photocurrent response and electrochemical impedance spectroscopy (EIS) of the samples were performed in a standard three-electrode cell using 0.1 M Na_2_SO_4_ solution as an electrolyte on an electrochemical workstation (CHI, 760E). An Ag/AgCl electrode was employed as the reference electrode, the Pt wires worked as the counter electrode, and the FTO coated with the samples (1 cm × 1 cm) acted as the working electrodes. Light was provided with an AM 1.5 G solar simulator (CEL-HXF300, Ceaulight).

### 4.5. Photocatalytic Activity Test

Photocatalytic activities were investigated with respect to the decomposition of RhB and TC under stimulating sunlight. The process was designed according to standard photocatalytic degradation tests: 20 mg photocatalyst was added into 50 mg/L aqueous suspensions of TC or RhB. Before the experiments, the suspensions were magnetically stirred in the dark for 30 min to ensure the establishment of a desorption–adsorption equilibrium between the samples and organic pollutants. Then, the suspensions were placed in a quartz test tube and degraded under simulated sunlight (AM 1.5). A 5 mL sample of the reaction solution was obtained every 10 min for TC and RhB. The suspension was centrifuged and then analyzed by a UV–vis spectrophotometer (HITACHI U–3900, Tokyo, Japan). Finally, the concentrations of RhB or TC were evaluated by measuring the absorption at 554 nm and 355 nm, respectively. The degradation efficiency *η* was evaluated using the following formula: *η = (A_t_ − A*_0_*)/A*_0_, where *A*_0_ and *A_t_* refer to the initial absorption of the aqueous contaminant and the absorption after degradation, respectively. Further, to show the photocatalytic performance of the composites, the apparent reaction rate constant (*k*, min^−1^) vs irradiation time is calculated by the pseudo-first-order rate equation model: *k = −ln(c/c*_0_*)/t*.

### 4.6. Quenching Experiments

The quenching experiments were performed using the same procedure as used in the photodegradation tests, except for the use of different trapping agents. Different radical scavengers were used, including isopropanol (IPA) as a scavenger for hydroxyl radical (•OH), L-ascorbic acid (LAA) as a scavenger for superoxide radical (•O_2_^−^), ethylene diamine tetraacetic acid disodium salt (EDTA-2Na) as a scavenger for h^+^. The method was similar to that of the photo-catalytic activity test, with the addition of 1 mM of quencher instead of RhB or TC.

## 5. Conclusions

The semiconductor and up-conversion concepts are combined and utilize a broadband spectrum (visible to NIR) to enhance photocatalytic activity. A novel Nd^3+^, Er^3+^@ NaYF_4_-BiOBr hybrid material is successfully synthesized through a simple two-step method. A micro-hexagonal up-conversion material, NE@NYF, was prepared by the hydrothermal method, and BiOBr nanosheets were loaded on the surface of NE@NYF through the in situ surface, self-grown strategy. The as-obtained photocatalyst exhibits broadband absorption, excellent electron transfer capability and increased photocatalytic performance. More importantly, the photocatalytic efficiency and apparent reaction rate constant of the NE@NYF-B composite were improved by 212% and 168%, respectively. The improved activity is ascribed to the synergistic effect of the semiconductor and up-conversion effects, which contribute to efficient charge–energy transition and improved separation of photogenerated electrons and holes. The 20% NE@NYF-B sample decomposed approximately 98.9% of RhB in 60 min and approximately 72.7% of TC in 10 h. Furthermore, based on the results obtained, the mechanisms of degradation of different pollutants were investigated. Importantly, this work infers that it is feasible to combine up-conversion materials for photodegradation, and it also provides a new NIR light utilization strategy of photon conversion.

## Figures and Tables

**Table 1:** Photocatalytic degradation performance of previous works.

Materials	Concentration Targets	Efficiency	Removal of TOC	Time	Ref.
NYF@C@Ag_3_PO_4_	10 mg/L CIP	69.64%	/	18 h	[11]
OVs-Bi/BiOBr/Sr_2_LaF_7_:Yb^3+^, Er^3+^	10 mg/L BPA	81%	/	180 min	[21]
UCNPs/g-C_3_N_4_	30 μM MO	~70%	/	60 min	[39]
UCN@SiO_2_@ZnO	5 mg/L RhB	61.2%	/	250 min	[40]
UCNP@TiO_2_	20 μg/L RhB	~98%	66%	300 min	[41]
CuWO_4_/BiOBr:Yb^3+^,Er^3^	20 mg/L TC	93.9%	/	60 min	[42]
N-CDs/BiOBr	10 mg/L RhB	89.3%	/	50 min	[43]
Nd^3+^, Er^3+^@NYF-BiOBr	50 mg/L RhB 50 mg/L TC	98.9% 97.2%	72.7% 74.6%	60 min	This work

## Data Availability

The data presented in this study are available on Supplementary Material.

## Supplementary Materials

The following supporting information can be downloaded at: https://www.mdpi.com/article/10.3390/molecules28186674/s1, Reference [44] are cited in the supplementary materials.
