# Supplementary material for: In Situ Construction of Near-Infrared Response Hybrid Up-Conversion Photocatalyst for Degrading Organic Dyes and Antibiotics
Source: Molecules. 2023 Sep 18;28(18):6674. doi: 10.3390/molecules28186674 (PMC10534851; doi:10.3390/molecules28186674)
Supplement: Supplementary file 1 [file molecules-28-06674-s001.zip › molecules-2523529-supplementary.pdf]

Supporting Information

In Situ Construction of Near-Infrared Response Hybrid Up-Conversion Photocatalyst for Degrading Organic Dyes and Antibiotics

Lianqing Yu [1,*,†], Yankun Wang [1,†], Xinhai Su [2], Chong Liu [1], Kehui Xue [1], Huihua Luo [1], Yaping Zhang [3] and Haifeng Zhu [3]

[1] School of Materials Science and Engineering, China University of Petroleum, Qingdao 266580, China; wang_yankun1@upc.edu.cn (Y.W.); buctlc96@163.com (C.L.); kehuixue@163.com (K.X.); huihua_luo@upc.edu.cn (H.L.)

[2] School of Chemical and Environmental Engineering, Hanshan Normal University, Guangdong 521041, China; suxinhai1@hstc.edu.cn

[3] College of Science, China University of Petroleum, Qingdao 266580, China; zhangyp@upc.edu.cn (Y.Z.); zhufeng_97@upc.edu.cn (H.Z.)

* Correspondence: yulq@upc.edu.cn

† These authors contributed equally to this work.

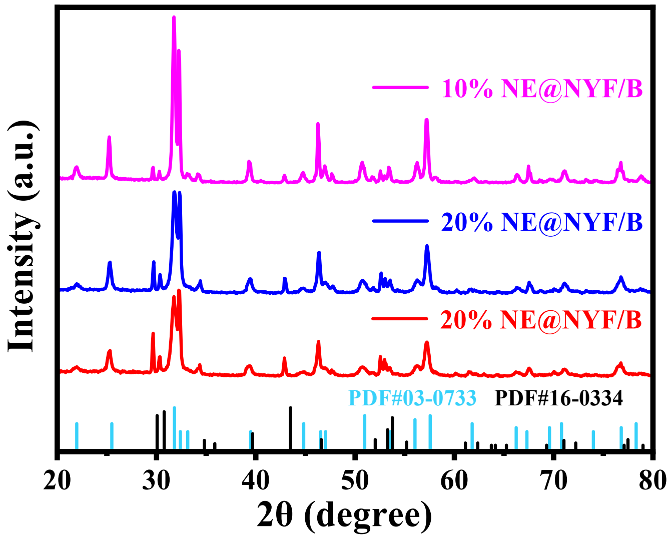

Figure S1. XRD patterns of NE@NYF-B samples with different ratios after the stability test.

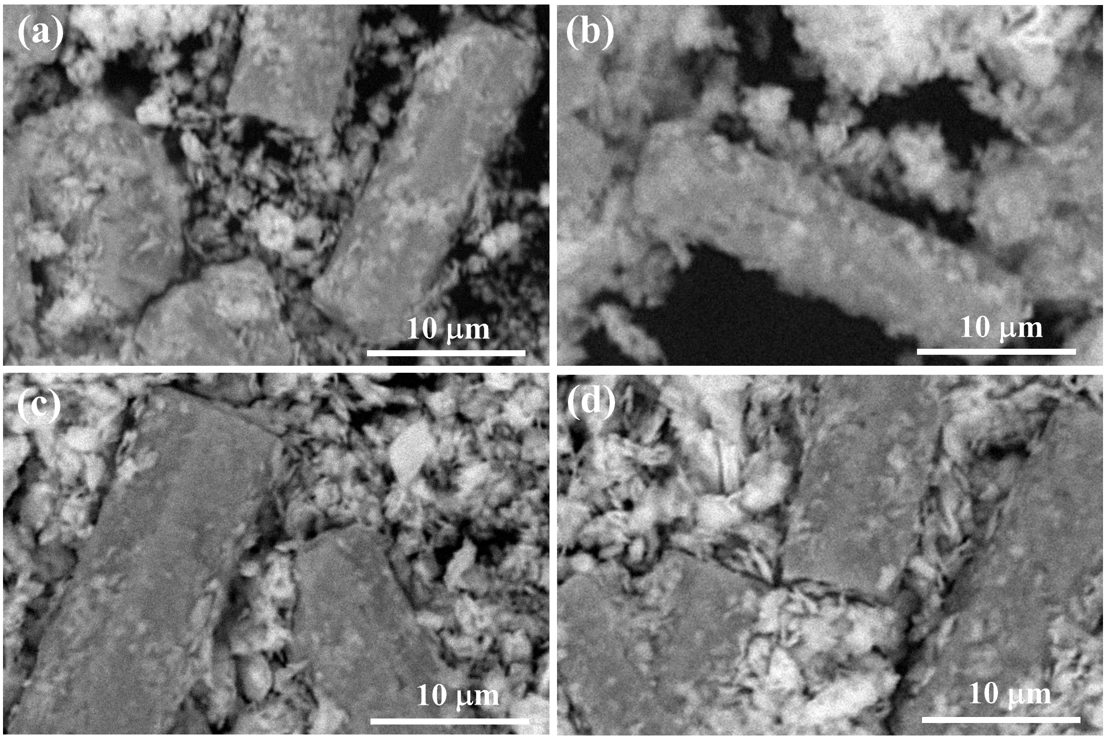

Figure S2. SEM image of (a) 5% NE@NYF-B, (b) 10% NE@NYF-B, (c) 20% NE@NYF-B, (d) 30% NE@NYF-B.

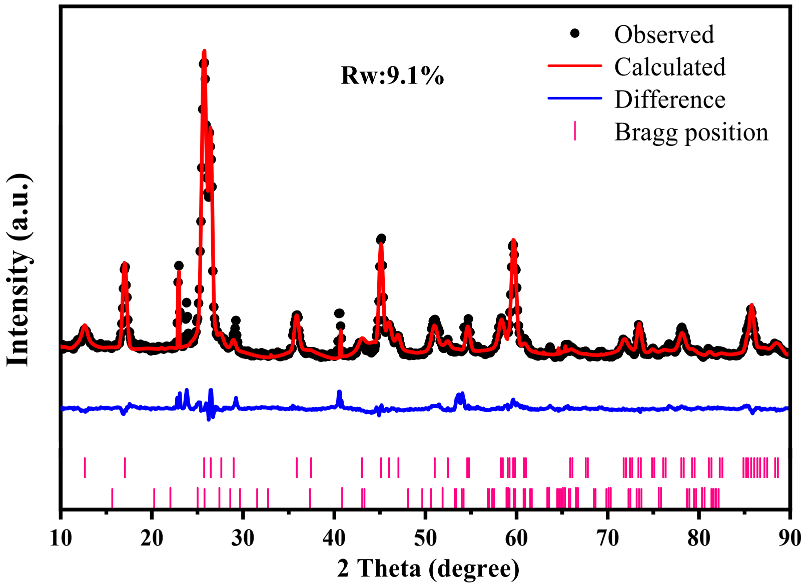

Figure S3. Rietveld refinement of XRD patterns for NE@NYF-B samples.

The XRD patterns of the samples were fitted by the Rietveld refinement method [1], and the results are shown in Figure S3. The results of the fit show 85.2% of BiOBr and 14.8% of NE@NYF.

Reference

1. Toby, B. H., EXPGUI, a graphical user interface for GSAS. *Journal of Applied Crystallography* **2001,** 34, (2), 210-213.
